# An analysis of stem cell training programs for physicians in the US—A mirage of credibility

**DOI:** 10.1016/j.stemcr.2025.102510

**Published:** 2025-05-29

**Authors:** Luqman Ellythy, Mohamed Addani, Zubin Master

**Affiliations:** 1 Mayo Clinic Alix School of Medicine, Mayo Clinic, Rochester, MN, USA; 2 Clinical and Translational Science, Mayo Clinic Graduate School of Biomedical Sciences, Mayo Clinic, Rochester, MN, USA; 3 Department of Social Sciences and Health Policy, Division of Public Health Sciences, Wake Forest University School of Medicine, Winston-Salem, NC, USA; 4 Wake Forest Institute of Regenerative Medicine, Wake Forest University School of Medicine, Winston-Salem, NC, USA; 5 Maya Angelou Center for Healthy Communities, Wake Forest University School of Medicine, Winston-Salem, NC, USA; 6 Center for Bioethics, Health & Society, Wake Forest University, Winston-Salem, NC, USA

## Abstract

Analyzing stem cell training businesses for United States (US) physicians shows the use of sensationalized marketing language emphasizing profits and growth with many of the instructors affiliated with clinics providing unproven therapies. We argue that many current pedagogical offerings for physicians interested in stem cell interventions are likely to offer questionable training and outline red flags for physicians interested in stem cell therapies.

## Introduction

The unproven stem cell intervention (SCI) industry refers primarily to an online, direct-to-consumer international market targeting mostly older adults with chronic, untreatable conditions, including neurodegenerative disorders, chronic pain, autoimmune disorders, diabetes, and arthritis, and aging indications. SCIs targeting such conditions should not be offered as part of routine care due to either scarcity or conflicting scientific evidence. Clinic advertisements are replete with misinformation and skewed language, omit risk information, and exclude language necessitating the need for regulatory and ethics oversight (Smith et al., 2021). Although most practicing physicians share concerns about the scientific uncertainty, medical and economic harm to patients, and misleading marketing (Smith et al., 2021), many also administer and recommend unproven SCIs to patients, most of whom are unqualified to provide the types of therapies for specific conditions (Fu et al., 2019). Despite regulatory scrutiny, there has been a 4.5-fold growth in the number of United States (US) clinics offering unproven SCIs since 2016 (Turner, 2021).

The growth in the number of providers and clinics offering unproven SCIs is of significant concern. While the concept of training physicians has been proposed over a decade ago (Knoepfler, 2013), some clinics advertise training opportunities for physicians as a token of legitimacy to incorporate unproven SCIs and anti-aging therapies into their practice (Sipp et al., 2017). In the US, continuing medical education (CME) is one of the main methods by which physicians maintain current knowledge and is overseen by the Accreditation Council for Continuing Medical Education. Legitimate regenerative science education within the US includes training during medical school, clinical fellowships offered by medical departments, and courses offered by professional bodies. Some cell-based therapy fellowships for physicians are available for specific subspecialties including hematology-oncology, ophthalmology, and pathology (UCSF; University of Pennsylvania Perelman School of Medicine Pathology and Laboratory Medicine; University of Illinois College of Medicine Department of Ophthalmology and Visual Sciences; USC Kerk School of Medicine, 2024). Professional bodies and societies have developed or are developing stem cell and regenerative medicine training tailored to clinicians (American Board of Regenerative Medicine; International Society for Stem Cell Research, 2024). Additionally, there are legitimate cell-based training programs focused on medical students (Wyles et al., 2019). Despite such notable efforts, offering potentially questionable quality training presents a major problem as clinicians considering a stem cell course may not understand the current state of stem cell science or believe SCIs are approved and ready for market. Moreover, physician learners who inadvertently offer unproven SCIs may face scrutiny by regulatory enforcement agencies and potential liability if an unproven SCI harms a patient. Finally, low-quality stem cell training provides a cover of legitimacy to some physicians to continue to offer unproven SCIs. To date, no research has yet to investigate the nature of suspicious cell-based training programs targeting clinicians and assess their adequacy.

To better understand the stem cell training landscape for physicians, we undertook a content analysis of US stem cell courses advertised on the internet and analyzed the nature of the training, advertisements, and the backgrounds of instructors listed among the training programs. Our focus was restricted to examining courses that aimed to promote the practice of unproven SCIs for practicing physicians, and we excluded stem cell training for medical students, residency or fellowship training in university-affiliated hospitals and academic medical centers, and those offered by reputable scientific and professional organizations (see supplemental information).

### Stem cell pedagogical offerings for physicians

We conducted Google searches using keywords and included onsite, online, or hybrid training programs targeting physicians or other clinicians on August, 2022 (see supplemental information). We systematically examined all information by companies offering stem cell training courses and identified 14 courses (6 onsite, 4 online, and 4 hybrid) that were offered by 11 different businesses. Courses ranged from single-day workshops to year-long programs, and the average cost was $4,919 ranging from $500 to $22,000. Nine courses listed a practicum component, most of which were procedural, including extraction and reintroduction of lipoaspirate; isolation and injection of platelet-rich plasma, lipoaspirate, and bone marrow aspirate-derived products; cadaver labs; ultrasound; esthetic procedures; and IV vitamin wellness. Ten courses listed 63 affiliate clinics, companies, and non-profit institutions (e.g., Cellular Hope Institute, BioTrend, HeartMD Institute, The Foundation for Alternative and Integrative Medicine, and Vita NOVAS), and four courses listed 9 universities (e.g., Columbia University, Sharda University, Atlantic International University, Rosalind Franklin University, University of South Florida, and University of Nevada Las Vegas) (see supplemental information).

Among the 9 businesses evaluated, 5 mentioned providing CME credit for their course(s). Additionally, 4 businesses stated using autologous cell sources as part of their training, 3 stated using both autologous and allogeneic cell sources, and 3 made no mention of cell sources.

### Marketing strategies to become a stem cell doctor

We analyzed marketing language among training organizations. Six training organizations mentioned they would assist learners in marketing and growing their practice with stem cell therapies and/or increasing revenue (Table 1). Eight organizations included learner testimonials as text and/or video, most of which were created by physicians, and all expressed a positive tone.

**Table 1:** Example quotes of marketing and revenue-generating language

Business development through website, email, and social media marketing programs. We provide programs and guidance on how to develop your practice through the most effective and efficient vehicles in your specific community. We will help you get the word out, let your community know what you are doing. The scope of the program is extensive. You choose the range and support needed. We offer a free Strategic Planning worksheet to everyone who wants to see where they are before starting any program. (Business #1)
This course is designed for all providers desiring an education in the practical techniques for offering regenerative therapies. This includes a basic understanding of the science of regenerative biologics, how to market and “sell” the procedures, and how to perform the procedures for musculoskeletal, cosmetic and systemic treatments. (Business #2)
Provide healthcare professionals with a full complement of medical training and resources that combine the science and art of Aesthetics, Anti-Aging and Pain Management Medicine to grow their business and increase revenue. (Business #3)
Procedures are typically cash based and costs $600–$800 per patient (Business #3)
Enhance patient care and realize the benefit of using and offering these regenerative therapies within your practice and create new revenue streams and greater patient satisfaction. (Business #3)
To assist serious Investors physicians and existing Healthcare Providers (Doctors, Hospitals, Clinics, Medical Centers) acquire and make proper use of our SVF deployment skills we have developed since 2010 and to opening their “American Center for Regenerative Medicine and Stem Cell Therapy” in their countries. (Business #4)
This workshop is tailor-made for practitioners looking to transition to a cash-pay business model and/or for those determined to take their practice to the next level in 2022. Attendees will learn the most effective, high-impact strategies in marketing, business management, social media, finance, leadership, and legal compliance. (Business #5)
Practice marketing, branding & business development training session. (Business #6)

### Instructor credentials and analysis of affiliated clinics

Eight courses (57%) among 7 organizations listed 172 instructors ranging from 4 to 71 instructors per course. Of the 172 instructors, 115 were physicians (MD and DO) with other instructors including doctor of acupuncture and oriental medicine, nurse practitioners, dentists, naturopathic doctors, dieticians and nutritionists, pharmacists, industry representatives, Drug Enforcement Administration inspectors, and medical coders. Specialties of physicians are reported in Table S2 (supplemental information).

Of the 115 physician instructors, we were able to identify 92 affiliated clinics. Using Google searches, we were able to determine whether the clinic affiliated with the instructor offered unproven, questionable, or proven treatments that were verified by a second coder. Unproven therapies were defined as lacking tier 1 clinical data to demonstrate clinical efficacy of the product, had expert opinion(s) suggesting the therapy should not be offered, and/or a Food and Drug Administration (FDA) warning. Questionable therapies had conflicting clinical data on treatment safety and/or efficacy and lacked consensus among scientific experts as to whether the therapy should be offered. Clinics offering both questionable and unproven interventions were coded as providing unproven therapies. Proven therapies were current standard of care or had FDA approval (see supplemental information).

**Figure 1:**
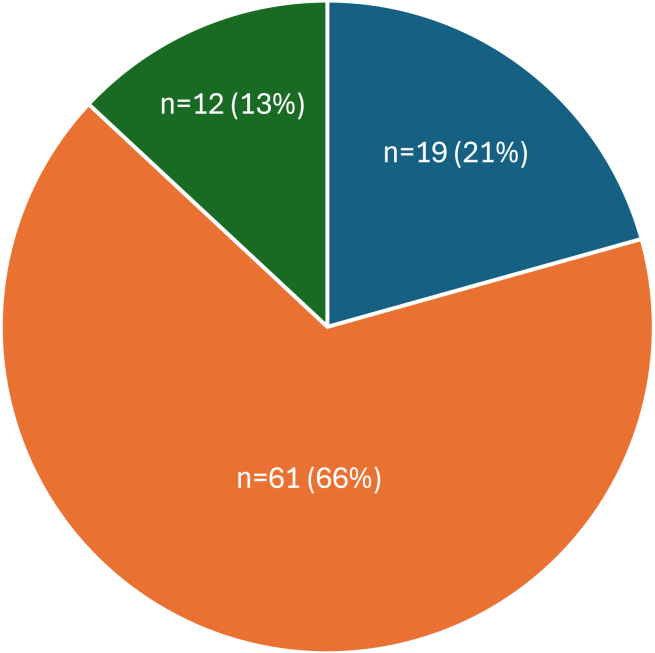
Instructor-affiliated clinics providing unproven, questionable, and proven treatments A total of 92 instructor-affiliated clinics were identified. Unproven therapies, orange; questionable therapies, green; proven therapies, dark blue.

Among the 92 instructor-affiliated clinics, most clinics (61, 66%) offered unproven therapies (e.g., intravenous vitamins, ozone therapy, platelet-rich plasma for autism) and 12 clinics (13%) offered questionable therapies (e.g., bioidentical hormone replacement therapy). Only 19 clinics (24%) offered proven therapies (Figure 1). Among clinics offering 1 or more unproven therapies, almost half (29, 48%) provided 3–5 unproven therapies, 21 clinics (35%) provided 1–2 unproven therapies, and 10 clinics (17%) provided 6 or more unproven therapies.

### Red flags for physicians considering stem cell training

At present, there is limited training available for physicians interested in stem cell and regenerative therapy. Our analysis suggests that the training being offered might present a false impression to learners that stem cells “can” be incorporated into clinical practice. This could have significant downstream effects in perpetuating misinformation about unproven SCIs and potentially harming patients, thus contributing to the already decreasing trust of physicians and the medical establishment. Our data suggest that illegitimate stem cell training programs use similar strategies to clinics engaged in the direct-to-consumer marketing of unproven SCIs in an attempt to showcase legitimacy of their training program. Practitioners may use such courses as tokens of scientific legitimacy to provide unproven SCIs to patients (Sipp et al., 2017). Physicians interested in learning more about stem cells and their potential incorporation into the clinic should aim to identify reputable training programs and look out for red flags. We highlight five red flags based on our data that physicians should reflect on when considering a stem cell training program.

The first red flag is to avoid training programs that use hyped language that promote the idea that SCIs can currently be incorporated into medical practice and that the learner would increase their revenue. This language could be seen as part of online marketing or among learner testimonials. For example, some programs use language that, upon completion of the training, the learner would be able to deliver SCIs to patients and no additional training is necessary or that physicians can be permitted to offer SCIs and they just need to be trained on how to do them. This message clearly conveyed by multiple national and international scientific bodies is that stem cell science is in preclinical or clinical phases of research and should not be offered to patients outside of a clinical trial (Lovell-Badge et al., 2021; Ikonomou et al., 2023).

A second red flag is having a narrow scope of training in an education program. While it is beyond the scope of this article to outline key topics for training physicians, it is likely that training medical professionals would cover a breadth of topics including stem cell biology, responsible clinical translation, regulations and policy, bioethics, patient communication, and clinical practice (Wyles et al., 2019). Courses focusing solely on stem cell therapy for practical application and providing practicums on how to administer stem cells or other unproven interventions should be met with suspicion.

A third red flag relates to the qualifications of the instructors. Our results showed that most instructors were physicians who may not have had the specialty training to treat specific degenerative conditions as previously shown (Fu et al., 2019) or to deliver the diversity of topics necessary for a stem cell training program for clinicians. A well-structured course for medical professionals that covers a breadth of topics discussed earlier should have instructors with diverse academic backgrounds (e.g., bioethics, regulatory, business management, and clinical trial expertise) who should be affiliated with an academic institution and have the appropriate expertise and experience to offer specific pedagogy. Potential learners should be weary of courses where the content focuses only on clinical application without rigorous evidence.

A fourth red flag for learners to consider is the reputability of the organizations providing stem cell courses. It is likely that legitimate training would be offered as part of an academic residency program, medical or graduate school, or reputable specialized professional or medical organizations (i.e., a clinical society), but this is not a straightforward marker of legitimacy. Several aforementioned businesses had affiliated themselves by displaying logos of recognized universities as a token of legitimacy that is likely to confuse potential learners about the credibility of such programs. Additionally, while some training programs claimed to be “fellowships,” only few were affiliated with an academic institution or a recognized academic society, and the extent of these bodies’ involvement in the course is unclear. Interested learners should seek out training in the handful of academic institutions that explicitly delineate proven and unproven therapies and have ongoing preclinical and clinical SCI trials (Wyles et al., 2019).

A fifth and final red flag is that just because courses offer CME credit does not mean they are legitimate as our results showed that 5 of 9 businesses provided CME courses. At least 10% of CME courses are industry sponsored and could lead to significant bias in training physicians (Ranganathan and Prasad, 2023).

## Conclusion

Physicians interested in learning more about stem cells and eventually incorporating them into practice need to be trained appropriately. Stem cell education courses for physicians in the US had marketing and revenue-generating language, and most instructors were linked to clinics administering unproven therapies.

It is unclear whether physicians interested in learning more about SCIs would be able to recognize legitimate from less legitimate educational courses and vendors easily. Our results suggest that physicians interested in training on SCIs should remain skeptical of courses using hyped language such as advertising monetary gains and the immediate incorporation of SCIs into practice. Learners should also remain skeptical of programs that do not train physicians broadly about stem cell topics, where the instructor pool is mostly of a single background, e.g., only clinicians, and where no research is conducted by instructors or participants. Finally, physicians should consider training offered by reputable medical and academic organizations.

## Author contributions

L.E. made substantial contributions to the collection and analysis of data, including identifying educational programs and searching the backgrounds of instructors, codebook development and modification, and served as primary qualitative coder. L.E. helped with the inital drafting of the paper, and revised the manuscript for important intellectual content. M.A. made substantial contributions to the analysis of data serving as secondary coder and revised the manuscript for important intellectual content. Z.M. made substantial contributions to the conception and design of the research project, helped revise the codebook, wrote the initial draft of the paper, and subsequently revised it for important intellectual content based on reviewer feedback. All authors approved the final version to be published and agreed to be accountable for all aspects of the work.

## Declaration of interests

Z.M. is a member of the Education Committee and the Public Policy Committee of the International Society for Stem Cell Research (ISSCR). ISSCR did not play a role in the research, and the views expressed in this paper do not reflect those of ISSCR, Wake Forest University School of Medicine, Wake Forest University, and Mayo Clinic.

## Supplemental information

Document S1. Tables S1 and S2 and supplemental methods

Document S2. Article plus supplemental information
