# Supplementary material for: An analysis of stem cell training programs for physicians in the US—A mirage of credibility
Source: Stem Cell Reports. 2025 May 29;20(6):102510. doi: 10.1016/j.stemcr.2025.102510 (PMC12181954; doi:10.1016/j.stemcr.2025.102510)
Supplement: Document S1. Tables S1 and S2 and supplemental methods [file mmc1.pdf]

Stem Cell Reports, Volume *20*

Supplemental Information

An analysis of stem cell training programs for physicians in the US—A mirage of credibility

Luqman Ellythy, Mohamed Addani, and Zubin Master

SUPPLEMENTAL INFORMATION

Stem Cell Reports 2025

An Analysis of Stem Cell Training Programs for Physicians in the U.S. – A Mirage of Credibility

Luqman Ellythy, Mohamed Addani, Zubin Master

Supplemental Methods

A google search of stem cell therapy educational courses for physicians was conducted in August 2022 using the terms “physician” or “doctor,” and “course” or “class” or “training,” and “stem cell therapy” or “regenerative medicine.” We included training websites offering onsite, online, or hybrid training targeted to physicians or other clinicians. Our selection of training programs targeted practicing physicians or other clinicians where the course aimed to promote the practice of unproven stem cell intervention (SCIs). We excluded training websites that were not written in English, where the primary target was not practicing physicians or other clinicians, and programs with only onsite training that was not offered in the U.S. These criteria excluded MS and PhD programs surrounding stem cell laboratory or clinical research. We excluded residency and fellowship programs offered by university-affiliated hospitals and academic medical centers, and training offered by reputable clinical societies and other professional bodies. All course websites meeting the inclusion criteria were systematically analyzed by a single coder using the codebook below. A second coder validated the primary coder’s data. Any discrepancies among coders were discussed and a mutual decision was reached.

Codebook Development:

The codebook was developed to analyze the nature of regenerative medicine training for physicians and other clinicians. The codebook was developed based on previously conducted research and inductively, based on an analysis of 3 training programs. The codebook was modified after inductive assessment of the 3 training programs and then systematically applied to all training websites meeting the inclusion criteria. The codebook contained 4 parts: 1) course features; 2) marketing language; and 3) instructors and their affiliations.

Final Codebook

1) Marketing Language:

We analyzed all websites within the domain of the training program for marketing language excluding learner testimonials.

- Included major themes related to the mission statement as a qualitative description and record exact quotes as examples
- Examples of hyped marketing language include: helping incorporate stem cells in practice, increase clinic profitability

2) Course Content

We collected course type, course name, number of hours, location, cost, type of training offered (e.g., regenerative medicine, aesthetic, marketing), mode of training (online, hybrid, or onsite), practicum description (if applicable), affiliation with external organizations (e.g., clinics, academic institutions), subject topics covered, mission statement, and instructor and learner testimonials.

Hours: Is the number of hours needed to complete the course indicated? (Y/N)

- If yes, include number of hours or describe the length of the course generally i.e., days, weeks, year.
- If yes, note the general timeframe given. If no, note if it is an online course to be taken at your own pace.

Mode of Training: Indicate the mode of training for this course: Online, Hybrid, or In-Person

Practicum: Does the course have a practicum (Y/N)?

- If yes, include a qualitative description of practicum

Onsite Location: Does the course have an onsite training component? (Y/N)

- If yes, include a qualitative description of the location of the onsite component

Cost: Is the cost of the course indicated without needing to sign up or email for more information? (Y/N)

- If yes, indicate the total cost of course

Continuing Medical Education (CME) Accreditation: Did the business mention having CME accreditation? Y/N)

Use of Autologous/Allogenic Cell Sources: Did the business mention anywhere that learners will receive training with autologous cell sources, allogenic cell sources, both, or did not mention?

Organization/Business Who Offers the Course(s): Include a qualitative description of the organization or business offering the course

- This may be the name of course itself
- Examine "About Us" statement, or objective(s) of the training program

Affiliations: Did the course list affiliated external partner(s)? (Y/N)

- If yes, provide a qualitative description of all listed affiliations
- Affiliated partners may include another business, organization, biotech company, universities, or academic institutions

Topics: Are the topics/subjects of the course covered?

- If yes, include a qualitative description of all the major topics/subjects covered

Outside References: Was there any statement/inference to an outside institution that provides stem cell treatment? (Y/N)

- Exclude instructor biographies
- If yes, include the name of the institution e.g., Mayo Clinic, name of unproven stem cell clinic

Learner Testimonials: Are there testimonials present on the website page? (Y/N)

- If yes, what format are they in? (Written, Videos or both)

3) Nature of Therapies Provided by Instructors at Their Affiliated Clinics:
For courses listing the names of instructors, we cross referenced the names with outside clinics via Google searches to evaluate the types of therapies instructors offered. We used the term “stem cell” with names to help identify instructors. In cases where additional clinic affiliation of the instructor was provided on the course website, we also searched the name of the instructor with the name of the clinic. We also cross-referenced the instructor’s name with the geographical location of their clinic if mentioned in their biography. We evaluated each instructor in terms of whether they provided treatments categorized as *unproven*, *questionable*, or *proven*.

Therapies classified as *unproven* were those that did not have any randomized controlled trials demonstrating treatment efficacy and lacked Tier 1 clinical data. If clinical studies were performed, we checked UpToDate, specialty guidelines from relevant clinical societies, and for the presence of an FDA warning to indicate whether the therapy should or should not be offered. Therapies classified as *questionable* included those with conflicting clinical data of treatment safety and/or efficacy and expert opinions in the academic literature were inconclusive as to whether the therapy should be offered. No clinic websites explicitly stated that the *unproven* or *questionable* therapies were given as part of a clinical trial, and thus *unproven* and *questionable* therapies were coded as “unproven” or questionable” respectively based on the above criteria. While websites did not explicitly mention whether *unproven* or *questionable* therapies were part of clinical research, this assumption constitutes a limitation of website content analysis. Therapies classified as *proven* only offered standard-of-care treatments or FDA approved treatments. Clinics classified as offering only *proven* therapies cannot advertise offering *unproven* or *questionable* therapies. A clinic would be counted as offering only *questionable* therapies if they offered no *unproven* therapies. Clinics offering both *questionable* and *unproven* therapies would be coded as providing *unproven*. The *unproven*, *questionable*, and *proven* categories were classified to clinics because more than 1 instructor (or other practitioner) was associated with a single clinic and we cannot be certain who among them was offering the *unproven*, *questionable*, or *proven* therapies (Fu et al., 2019 JAMA). A physician trainee (LE) determined whether therapies were classified as unproven, questionable, or proven and sought advice from senior Mayo Clinic physician experts as needed.

Are the instructors listed on the website and clearly indicated as instructor or team? (Y/N)

- If yes list the number of instructors
- Identify the backgrounds of all instructors
- For physician instructors with MD or DO, identify areas of residency or fellowship specialty training.

Unproven & Questionable Therapies:

- For each instructor-affiliated clinic, write down the number of unproven and questionable therapies provided for specific indications. Application of an unproven/questionable therapy for each indication listed is counted except IV vitamins (see Table S1)

Proven Therapies:
There is no evidence of the instructor providing unproven or questionable therapies in affiliated clinic.

- Counted as 0 for the number of unproven therapies provided.

Table S1: Level of evidence for unproven and questionable therapies offered at instructor-affiliated clinics

Treatment	Number of Clinics	Indication	Classification	References
Hyperbaric Oxygen	5	Anti-aging	Unproven	1
Ozone	7	Anti-aging	Unproven	2
Bioidentical Hormone Replacement Therapy	18	Anti-aging, increased energy	Questionable	3,4
NAD+ IV therapy	2	Anti-aging	Unproven	5,6
Vampire Needling + platelet-rich plasma (PRP) for face	20	Skin rejuvenation	Unproven	7
IV vitamins: Myer's cocktail, Curcumin, Vitamin C.	25	Antiaging, cancer therapy	Unproven	8, 9, 43
Infrared and red-light sauna	2	Energy and antiaging	Unproven	10, 39
Activated Air Therapy/singlet oxygen therapy	1	Biohacking (antiaging)	Unproven	11
Vitamin B12 injections	1	Weight loss	Unproven	12
VSELs (very small embryonic-like stem cells)	1	Decreased joint pain, increased energy, improved metabolic function, and improved cognitive function	Unproven	13
pH manipulation therapy	2	Cancer	Unproven	14
Prolotherapy	1	Chronic pain	Questionable	15, 16
Prolozone	6	Chronic neck and back pain + joint arthritis	Unproven	17
Therapeutic ultrasound	3	Healing Injuries. Improve pain, function and range of motion.	Unproven	18
Photoactivated PRP	1	"Healing"	Unproven	19
P shots (priapism shots)	11	Erectile Dysfunction	Unproven	20
O shots (orgasm shots)	10	Vaginal rejuvenation and sexual dysfunction	Unproven	21

HCG (human chorionic gonadotropin) diet	10	Weight loss	Unproven	22
NO therapy	1	Sexual dysfunction	Unproven	23
Total nonspecific PRP	10	No Indication	Unproven	
PRP	5	Autism	Unproven	24
PRP	8	MS	Unproven	25 26
PRP	9	COPD	Unproven	27 28 no RCTs
PRP	1	Heart Failure	Unproven	29
PRP	10	joints/arthritis	Questionable	31
TMS (transcranial magnetic stimulation)	1	Autism	Unproven	30
Stem cells	10	orthobiologics	Unproven	32, 33
Extracorporeal shock therapy	2	Tendinopathy	Questionable	33
Amniotic stem cells	3		Unproven	34
PRP	5	Hair restoration	Questionable	52
Thread embedding Therapy	5	Hair restoration	Unproven	7
Therapeutic plasma exchange	1	Anti-aging	Unproven	35
Homeopathy	1	Any specific indication	Unproven	47
Laser therapy	2	Tendinopathy	Unproven	36
Alpha 2 Macroglobulin (A2M) Injections	5	Ortho	Unproven	37
Peptide therapy	5	Cancer	Unproven	46
Tenex	10	Tendinopathy	Unproven	45
Reflexotherapy	1	cancer	Unproven	38
Whole body cryotherapy	1	Weight loss and fibromyalgia	Unproven	40, 41
Vaginal rejuvenation radiofrequency	1	Sexual Function	Unproven	42
Lonator body cleanse	1	Toxin removal	Unproven	44
IV ketamine	1	Wellness and fatigue	Unproven	49
Hydropathy	1	Pain	Unproven	50
Quantum Therapy	1	Pain	Unproven	51

Among the 115 physician instructors, we documented their specialty training (Table S2).

Table S2. Residency and Fellowship Specialties Among Physician Instructors

Specialty	Number of Physician Instructors
Aesthetics	1
Allergy and Immunology	1
Anesthesiology	5
Cardiology	4
Dermatology	6
Emergency Medicine	6
Endocrinology	1
Ear, Nose and Throat	2
Facial Plastics	2
Family Medicine	15
General Surgery	5
Geriatrics	1
Gynecology-Oncology	1
Internal Medicine	9
Neurology	2
Neurosurgery	2
Obstetrics and Gynecology	6
Occupational and Environmental Medicine	1
Oncology	2
Ophthalmology	1
Oral Maxillofacial Surgery	1
Orthopedics	4
Pain	3
Pathology	1
Pediatrics	2
Physical Medicine and Rehabilitation	2
Plastic Surgery	5
Preventative Medicine	1
Psychiatry	3
Radiology	1
Spine (Orthopedics)	1
Sports Medicine	14
Unknown	2
Urology	2

References:

1. Jones MW, Cooper JS. Hyperbaric Therapy for Wound Healing. [Updated 2023 Jun 12]. In: StatPearls [Internet]. Treasure Island (FL): StatPearls Publishing; 2024 Jan-. Available from: https://www.ncbi.nlm.nih.gov/books/NBK459172/
2. U.S. Food and Drug Administration. (n.d.). 21 CFR § 801.415 - Labeling for in vitro diagnostic devices. Retrieved August 13, 2024, from https://www.accessdata.fda.gov/scripts/cdrh/cfdocs/cfcfr/cfrsearch.cfm?fr=801.415
3. U.S. Food and Drug Administration. (n.d.). National Academies of Sciences, Engineering, and Medicine (NASEM) study on the clinical utility of treating patients. U.S. Food and Drug Administration. Retrieved August 13, 2024, from https://www.fda.gov/drugs/human-drug-compounding/national-academies-science-engineering-and-medicine-nasem-study-clinical-utility-treating-patients
4. National Academies of Sciences, Engineering, and Medicine. (2019). The clinical utility of treating patients with antimicrobial agents. In The clinical utility of treating patients with antimicrobial agents (Chapter 9). National Academies Press. https://nap.nationalacademies.org/read/25791/chapter/9#166
5. Braidy N, Liu Y. NAD+ therapy in age-related degenerative disorders: A benefit/risk analysis. Exp Gerontol. 2020 Apr;132:110831. doi: 10.1016/j.exger.2020.110831. Epub 2020 Jan 7. PMID: 31917996.
6. Radenkovic D, Reason, Verdin E. Clinical Evidence for Targeting NAD Therapeutically. Pharmaceuticals (Basel). 2020 Sep 15;13(9):247. doi: 10.3390/ph13090247. PMID: 32942582; PMCID: PMC7558103.
7. Gupta AK, Versteeg SG, Rapaport J, Hausauer AK, Shear NH, Piguet V. The Efficacy of Platelet-Rich Plasma in the Field of Hair Restoration and Facial Aesthetics-A Systematic Review and Meta-analysis. J Cutan Med Surg. 2019 Mar/Apr;23(2):185-203. doi: 10.1177/1203475418818073. Epub 2019 Jan 4. PMID: 30606055.
8. Moertel CG, Fleming TR, Creagan ET, Rubin J, O'Connell MJ, Ames MM. High-dose vitamin C versus placebo in the treatment of patients with advanced cancer who have had no prior chemotherapy. A randomized double-blind comparison. N Engl J Med. 1985 Jan 17;312(3):137-41. doi: 10.1056/NEJM198501173120301. PMID: 3880867.
9. Nelson, K. M., Dahlin, J. L., Bisson, J., Graham, J., Pauli, G. F., & Walters, M. A. (2017). The Essential Medicinal Chemistry of Curcumin. Journal of medicinal chemistry, 60(5), 1620–1637. https://doi.org/10.1021/acs.jmedchem.6b00975
10. Beever R. Far-infrared saunas for treatment of cardiovascular risk factors: summary of published evidence. Can Fam Physician. 2009 Jul;55(7):691-6. PMID: 19602651; PMCID: PMC2718593.
11. Li, X., Lovell, J. F., Yoon, J., & Chen, X. (2020). Clinical development and potential of photothermal and photodynamic therapies for cancer. Nature reviews. Clinical oncology, 17(11), 657–674. https://doi.org/10.1038/s41571-020-0410-2
12. Boachie, J., Adaikalakoteswari, A., Samavat, J., & Saravanan, P. (2020). Low Vitamin B12 and Lipid Metabolism: Evidence from Pre-Clinical and Clinical Studies. Nutrients, 12(7), 1925. https://doi.org/10.3390/nu12071925
13. Ratajczak, M. Z., Ratajczak, J., & Kucia, M. (2019). Very Small Embryonic-Like Stem Cells (VSELs). Circulation research, 124(2), 208–210. https://doi.org/10.1161/CIRCRESAHA.118.314287

14. 1Yang M, Zhong X, Yuan Y. Does Baking Soda Function as a Magic Bullet for Patients With Cancer? A Mini Review. Integrative Cancer Therapies. 2020;19. doi:10.1177/1534735420922579
15. Morath, O., Kubosch, E. J., Taeymans, J., Zwingmann, J., Konstantinidis, L., Südkamp, N. P., & Hirschmüller, A. (2018). The effect of sclerotherapy and prolotherapy on chronic painful Achilles tendinopathy-a systematic review including meta-analysis. Scandinavian journal of medicine & science in sports, 28(1), 4–15. https://doi.org/10.1111/sms.12898
16. Taheem, Y. and Suvar, T. (2021) Prolotherapy: Review with background history, mechanism of action, and current evidence, ASRA Pain Medicine. Available at: https://www.asra.com/news-publications/asra-updates/blog-landing/legacy-b-blog-posts/2021/10/15/prolotherapy-review-with-background-history-mechanism-of-action-and-current-evidence (Accessed: 13 August 2024).
17. CFR - Code of Federal Regulations Title 21 (no date) accessdata.fda.gov. Available at: https://www.accessdata.fda.gov/scripts/cdrh/cfdocs/cfcfr/cfrsearch.cfm?fr=801.415#:~:text=801.415%20Maximum%20acceptable%20level%20of,tolerated%20by%20man%20and%20animals. (Accessed: 13 August 2024).
18. Miller DL, Smith NB, Bailey MR, Czarnota GJ, Hynynen K, Makin IR; Bioeffects Committee of the American Institute of Ultrasound in Medicine. Overview of therapeutic ultrasound applications and safety considerations. J Ultrasound Med. 2012 Apr;31(4):623-34. doi: 10.7863/jum.2012.31.4.623. PMID: 22441920; PMCID: PMC3810427.
19. Paterson, K. L., Nicholls, M., Bennell, K. L., & Bates, D. (2016). Intra-articular injection of photo-activated platelet-rich plasma in patients with knee osteoarthritis: a double-blind, randomized controlled pilot study. BMC musculoskeletal disorders, 17, 67. https://doi.org/10.1186/s12891-016-0920-3
20. Burnett, A. L., Nehra, A., Breau, R. H., Culkin, D. J., Faraday, M. M., Hakim, L. S., Heidelbaugh, J., Khera, M., McVary, K. T., Miner, M. M., Nelson, C. J., Sadeghi-Nejad, H., Seftel, A. D., & Shindel, A. W. (2018). Erectile Dysfunction: AUA Guideline. The Journal of urology, 200(3), 633–641. https://doi.org/10.1016/j.juro.2018.05.004
21. Stanley, E. E., & Pope, R. J. (2022). Characteristics of Female Sexual Health Programs and Providers in the United States. Sexual medicine, 10(4), 100524. https://doi.org/10.1016/j.esxm.2022.100524
22. Commissioner, O. of the (2020) Avoid dangerous HCG diet products, U.S. Food and Drug Administration. Available at: https://www.fda.gov/consumers/consumer-updates/avoid-dangerous-hcg-diet-products (Accessed: 13 August 2024).
23. Burnett AL. The role of nitric oxide in erectile dysfunction: implications for medical therapy. J Clin Hypertens (Greenwich). 2006 Dec;8(12 Suppl 4):53-62. doi: 10.1111/j.1524-6175.2006.06026.x. PMID: 17170606; PMCID: PMC8109295.
24. Ardhanareeswaran K, Coppola G, Vaccarino F. The use of stem cells to study autism spectrum disorder. Yale J Biol Med. 2015 Mar 4;88(1):5-16. PMID: 25745370; PMCID: PMC4345539.
25. Farid MF, Abouelela YS, Yasin NAE, Mousa MR, Ibrahim MA, Prince A, Rizk H. A novel cell-free intrathecal approach with PRP for the treatment of spinal cord multiple sclerosis in cats. Inflamm Regen. 2022 Oct 14;42(1):45. doi: 10.1186/s41232-022-00230-w. PMID: 36229845; PMCID: PMC9563497.

26. Borhani-Haghighi, M., & Mohamadi, Y. (2019). The therapeutic effect of platelet-rich plasma on the experimental autoimmune encephalomyelitis mice. Journal of neuroimmunology, 333, 476958. https://doi.org/10.1016/j.jneuroim.2019.04.018
27. Mammoto T, Chen Z, Jiang A, Jiang E, Ingber DE, Mammoto A. Acceleration of Lung Regeneration by Platelet-Rich Plasma Extract through the Low-Density Lipoprotein Receptor-Related Protein 5-Tie2 Pathway. Am J Respir Cell Mol Biol. 2016 Jan;54(1):103-13. doi: 10.1165/rcmb.2015-0045OC. PMID: 26091161; PMCID: PMC5455682.
28. Knight AD, Kacker S. Platelet-Rich Plasma Treatment for Chronic Respiratory Disease. Cureus. 2023 Jan 2;15(1):e33265. doi: 10.7759/cureus.33265. PMID: 36741673; PMCID: PMC9891651.
29. Spartalis E, Tomos P, Moris D, Athanasiou A, Markakis C, Spartalis MD, Troupis T, Dimitroulis D, Perrea D. Role of platelet-rich plasma in ischemic heart disease: An update on the latest evidence. World J Cardiol. 2015 Oct 26;7(10):665-70. doi: 10.4330/wjc.v7.i10.665. PMID: 26516421; PMCID: PMC4620078.
30. Shen, Y. X., Fan, Z. H., Zhao, J. G., & Zhang, P. (2009). The application of platelet-rich plasma may be a novel treatment for central nervous system diseases. Medical hypotheses, 73(6), 1038–1040. https://doi.org/10.1016/j.mehy.2009.05.021
31. Weissman, L. and Harris, H.K. (2024) Autism spectrum disorder in children and adolescents: Complementary and alternative therapies, UpToDate. Available at: https://www.uptodate.com/contents/autism-spectrum-disorder-in-children-and-adolescents-complementary-and-alternative-therapies?search=MeRT+TMS&source=search_result&selectedTitle=10~150&usage_type=default&display_rank=10#H2917575028 (Accessed: 13 August 2024).
32. O'Dowd A. Update on the Use of Platelet-Rich Plasma Injections in the Management of Musculoskeletal Injuries: A Systematic Review of Studies From 2014 to 2021. Orthopaedic Journal of Sports Medicine. 2022;10(12). doi:10.1177/23259671221140888
33. Young, M, Dijkstra, P, Biologic therapies for tendon and muscle injury UpToDate. (2024). Retrieved August 13, 2024, from Uptodate.com website: https://www.uptodate.com/contents/biologic-therapies-for-tendon-and-muscle-injury?search=prgf&source=search_result&selectedTitle=1~65&usage_type=default&display_rank=1#H2827233597
34. Purdam, CR, UpToDate. (2024). Retrieved August 13, 2024, from Uptodate.com website: https://www.uptodate.com/contents/overuse-persistent-tendinopathy-overview-of-management#H3483872523
35. Suh, S., Yale, K. L., & Mesinkovska, N. A. (2021). 28383 The effectiveness of thread-embedding therapy for treating scarring and nonscarring alopecia. Journal of the American Academy of Dermatology, 85(3), AB186–AB186. https://doi.org/10.1016/j.jaad.2021.06.757
36. Hunter M. Use of homeopathy in NHS not justified. BMJ. 2002 Mar 9;324(7337):565. PMCID: PMC1172082.
37. Tumilty, S., Munn, J., McDonough, S., Hurley, D. A., Basford, J. R., & Baxter, G. D. (2010). Low level laser treatment of tendinopathy: a systematic review with meta-analysis. Photomedicine and laser surgery, 28(1), 3–16. https://doi.org/10.1089/pho.2008.2470
38. Vajapey, S., Ghenbot, S., Baria, M. R., Magnussen, R. A., & Vasileff, W. K. (2021). Utility of Percutaneous Ultrasonic Tenotomy for Tendinopathies: A Systematic Review. Sports health, 13(3), 258–264. https://doi.org/10.1177/1941738120951764

39. Ernst, E., P Posadzki, & Lee, M. S. (2011). Reflexology: An update of a systematic review of randomised clinical trials. Maturitas, 68(2), 116–120. https://doi.org/10.1016/j.maturitas.2010.10.011
40. Glass G. E. (2021). Photobiomodulation: The Clinical Applications of Low-Level Light Therapy. Aesthetic surgery journal, 41(6), 723–738. https://doi.org/10.1093/asj/sjab025
41. Hernández-Bule ML, Naharro-Rodríguez J, Bacci S, Fernández-Guarino M. Unlocking the Power of Light on the Skin: A Comprehensive Review on Photobiomodulation. Int J Mol Sci. 2024 Apr 19;25(8):4483. doi: 10.3390/ijms25084483. PMID: 38674067; PMCID: PMC11049838.
42. Whole body cryotherapy can be hazardous to your skin (no date) American Academy of Dermatology. Available at: https://www.aad.org/public/cosmetic/safety/cryotherapy (Accessed: 13 August 2024).
43. Commissioner, O. of the (no date) Statement from FDA commissioner Scott Gottlieb, M.D., on efforts to safeguard women's health from deceptive health claims and significant risks related to devices marketed for use in medical procedures for 'vaginal rejuvenation', U.S. Food and Drug Administration. Available at: https://www.fda.gov/news-events/press-announcements/statement-fda-commissioner-scott-gottlieb-md-efforts-safeguard-womens-health-deceptive-health-claims (Accessed: 13 August 2024).
44. Ali, A., Njike, V. Y., Northrup, V., Sabina, A. B., Williams, A. L., Liberti, L. S., Perlman, A. I., Adelson, H., & Katz, D. L. (2009). Intravenous micronutrient therapy (Myers' Cocktail) for fibromyalgia: a placebo-controlled pilot study. Journal of alternative and complementary medicine (New York, N.Y.), 15(3), 247–257. https://doi.org/10.1089/acm.2008.0410
45. Barman P, Joshi S, Sharma S, Preet S, Sharma S, Saini A. Strategic Approaches to Improvise Peptide Drugs as Next Generation Therapeutics. Int J Pept Res Ther. 2023;29(4):61. doi: 10.1007/s10989-023-10524-3. Epub 2023 May 24. PMID: 37251528; PMCID: PMC10206374.
46. Castro, J. C., Wang, D., & Chien, G. C. C. (2022). Regenerative medicine for neuropathic pain: physiology, ultrasound and therapies with a focus on alpha-2-macroglobulin. Pain management, 12(6), 779–793. https://doi.org/10.2217/pmt-2022-0006
47. Fernández-Zarzoso, M., Gómez-Seguí, I., & de la Rubia, J. (2019). Therapeutic plasma exchange: Review of current indications. Transfusion and apheresis science : official journal of the World Apheresis Association : official journal of the European Society for Haemapheresis, 58(3), 247–253. https://doi.org/10.1016/j.transci.2019.04.007
48. Center. (2024). Important Patient and Consumer Information About Regenerative Medicine. Retrieved August 14, 2024, from U.S. Food and Drug Administration website: https://www.fda.gov/vaccines-blood-biologics/consumers-biologics/important-patient-and-consumer-information-about-regenerative-medicine-therapies
49. Thase, M. and Connolly, R. (2024) Ketamine and esketamine for treating unipolar depression in adults: Administration, efficacy, and adverse effects, UpToDate. Available at: https://www.uptodate.com/contents/ketamine-and-esketamine-for-treating-unipolar-depression-in-adults-administration-efficacy-and-adverse-effects?search=ketamine&source=search_result&selectedTitle=2~150&usage_type=default&display_rank=1 (Accessed: 27 August 2024).
50. Ortiz M, Koch AK, Cramer H, Linde K, Rotter G, Teut M, Brinkhaus B, Haller H. Clinical effects of Kneipp hydrotherapy: a systematic review of randomised controlled trials. BMJ Open. 2023 Jul 9;13(7):e070951. doi: 10.1136/bmjopen-2022-070951. PMID: 37423627; PMCID: PMC10335435.

51. Barassi G, Pokorski M, Pellegrino R, Supplizi M, Prosperi L, Marinucci C, Di Simone E, Mariani C, Younes A, Di Iorio A. Quantum Medicine: A Role of Extremely Low-Frequency Magnetic Fields in the Management of Chronic Pain. Adv Exp Med Biol. 2022;1375:23-28. doi: 10.1007/5584_2021_697. PMID: 35038149.
52. U.S. Food & Drug Administration. 2021. Important patient and consumer information about regenerative medicine therapies. https://www.fda.gov/vaccines-blood-biologics/consumers-biologics/important-patient-and-consumer-information-about-regenerative-medicine-therapies.
