# Supplementary material for: An analysis of stem cell training programs for physicians in the US—A mirage of credibility
Source: Stem Cell Reports. 2025 May 29;20(6):102510. doi: 10.1016/j.stemcr.2025.102510 (PMC12181954; doi:10.1016/j.stemcr.2025.102510)
Supplement: Document S2. Article plus supplemental information [file mmc2.pdf]

Stem Cell Reports
Forum
OPEN ACCESS

An analysis of stem cell training programs for physicians in the US—A mirage of credibility

Luqman Ellythy,[1] Mohamed Addani,[2] and Zubin Master[3,4,5,6,*]
[1]Mayo Clinic Alix School of Medicine, Mayo Clinic, Rochester, MN, USA
[2]Clinical and Translational Science, Mayo Clinic Graduate School of Biomedical Sciences, Mayo Clinic, Rochester, MN, USA
[3]Department of Social Sciences and Health Policy, Division of Public Health Sciences, Wake Forest University School of Medicine, Winston-Salem, NC, USA
[4]Wake Forest Institute of Regenerative Medicine, Wake Forest University School of Medicine, Winston-Salem, NC, USA
[5]Maya Angelou Center for Healthy Communities, Wake Forest University School of Medicine, Winston-Salem, NC, USA
[6]Center for Bioethics, Health & Society, Wake Forest University, Winston-Salem, NC, USA
*Correspondence: zmaster@wakehealth.edu
https://doi.org/10.1016/j.stemcr.2025.102510

SUMMARY

Analyzing stem cell training businesses for United States (US) physicians shows the use of sensationalized marketing language emphasizing profits and growth with many of the instructors affiliated with clinics providing unproven therapies. We argue that many current pedagogical offerings for physicians interested in stem cell interventions are likely to offer questionable training and outline red flags for physicians interested in stem cell therapies.

INTRODUCTION

The unproven stem cell intervention (SCI) industry refers primarily to an online, direct-to-consumer international market targeting mostly older adults with chronic, untreatable conditions, including neurodegenerative disorders, chronic pain, autoimmune disorders, diabetes, and arthritis, and aging indications. SCIs targeting such conditions should not be offered as part of routine care due to either scarcity or conflicting scientific evidence. Clinic advertisements are replete with misinformation and skewed language, omit risk information, and exclude language necessitating the need for regulatory and ethics oversight (Smith et al., 2021). Although most practicing physicians share concerns about the scientific uncertainty, medical and economic harm to patients, and misleading marketing (Smith et al., 2021), many also administer and recommend unproven SCIs to patients, most of whom are unqualified to provide the types of therapies for specific conditions (Fu et al., 2019). Despite regulatory scrutiny, there has been a 4.5-fold growth in the number of United States (US) clinics offering unproven SCIs since 2016 (Turner, 2021).

The growth in the number of providers and clinics offering unproven SCIs is of significant concern. While the concept of training physicians has been proposed over a decade ago (Knoepfler, 2013), some clinics advertise training opportunities for physicians as a token of legitimacy to incorporate unproven SCIs and anti-aging therapies into their practice (Sipp et al., 2017). In the US, continuing medical education (CME) is one of the main methods by which physicians maintain current knowledge and is overseen by the Accreditation Council for Continuing Medical Education. Legitimate regenerative science education within the US includes training during medical school, clinical fellowships offered by medical departments, and courses offered by professional bodies. Some cell-based therapy fellowships for physicians are available for specific subspecialties including hematology-oncology, ophthalmology, and pathology (UCSF; University of Pennsylvania Perelman School of Medicine Pathology and Laboratory Medicine; University of Illinois College of Medicine Department of Ophthalmology and Visual Sciences; USC Kerk School of Medicine, 2024). Professional bodies and societies have developed or are developing stem cell and regenerative medicine training tailored to clinicians (American Board of Regenerative Medicine; International Society for Stem Cell Research, 2024). Additionally, there are legitimate cell-based training programs focused on medical students (Wyles et al., 2019). Despite such notable efforts, offering potentially questionable quality training presents a major problem as clinicians considering a stem cell course may not understand the current state of stem cell science or believe SCIs are approved and ready for market. Moreover, physician learners who inadvertently offer unproven SCIs may face scrutiny by regulatory enforcement agencies and potential liability if an unproven SCI harms a patient. Finally, low-quality stem cell training provides a cover of legitimacy to some physicians to continue to offer unproven SCIs. To date, no research has yet to investigate the nature of suspicious cell-based training programs targeting clinicians and assess their adequacy.

To better understand the stem cell training landscape for physicians, we undertook a content analysis of US stem cell courses advertised on the internet and analyzed the nature of the

© 2025 The Authors.
Published by Elsevier Inc. on behalf of International Society for Stem Cell Research.
This is an open access article under the CC BY license (http://creativecommons.org/licenses/by/4.0/).

Table 1. Example quotes of marketing and revenue-generating language

Business development through website, email, and social media marketing programs. We provide programs and guidance on how to develop your practice through the most effective and efficient vehicles in your specific community. We will help you get the word out, let your community know what you are doing. The scope of the program is extensive. You choose the range and support needed. We offer a free Strategic Planning worksheet to everyone who wants to see where they are before starting any program. (Business #1)
This course is designed for all providers desiring an education in the practical techniques for offering regenerative therapies. This includes a basic understanding of the science of regenerative biologics, how to market and "sell" the procedures, and how to perform the procedures for musculoskeletal, cosmetic and systemic treatments. (Business #2)
Provide healthcare professionals with a full complement of medical training and resources that combine the science and art of Aesthetics, Anti-Aging and Pain Management Medicine to grow their business and increase revenue. (Business #3)
Procedures are typically cash based and costs $600–$800 per patient (Business #3)
Enhance patient care and realize the benefit of using and offering these regenerative therapies within your practice and create new revenue streams and greater patient satisfaction. (Business #3)
To assist serious Investors physicians and existing Healthcare Providers (Doctors, Hospitals, Clinics, Medical Centers) acquire and make proper use of our SVF deployment skills we have developed since 2010 and to opening their "American Center for Regenerative Medicine and Stem Cell Therapy" in their countries. (Business #4)
This workshop is tailor-made for practitioners looking to transition to a cash-pay business model and/or for those determined to take their practice to the next level in 2022. Attendees will learn the most effective, high-impact strategies in marketing, business management, social media, finance, leadership, and legal compliance. (Business #5)
Practice marketing, branding & business development training session. (Business #6)

training, advertisements, and the backgrounds of instructors listed among the training programs. Our focus was restricted to examining courses that aimed to promote the practice of unproven SCIs for practicing physicians, and we excluded stem cell training for medical students, residency or fellowship training in university-affiliated hospitals and academic medical centers, and those offered by reputable scientific and professional organizations (see supplemental information).

Stem cell pedagogical offerings for physicians

We conducted Google searches using keywords and included onsite, online, or hybrid training programs targeting physicians or other clinicians on August, 2022 (see supplemental information). We systematically examined all information by companies offering stem cell training courses and identified 14 courses (6 onsite, 4 online, and 4 hybrid) that were offered by 11 different businesses. Courses ranged from single-day workshops to year-long programs, and the average cost was $4,919 ranging from $500 to $22,000. Nine courses listed a practicum component, most of which were procedural, including extraction and reintroduction of lipoaspirate; isolation and injection of platelet-rich plasma, lipoaspirate, and bone marrow aspirate-derived products; cadaver labs; ultrasound; esthetic procedures; and IV vitamin wellness. Ten courses listed 63 affiliate clinics, companies, and non-profit institutions (e.g., Cellular Hope Institute, BioTrend, HeartMD Institute, The Foundation for Alternative and Integrative Medicine, and Vita NOVAS), and four courses listed 9 universities (e.g., Columbia University, Sharda University, Atlantic International University, Rosalind Franklin University, University of South Florida, and University of Nevada Las Vegas) (see supplemental information).

Among the 9 businesses evaluated, 5 mentioned providing CME credit for their course(s). Additionally, 4 businesses stated using autologous cell sources as part of their training, 3 stated using both autologous and allogeneic cell sources, and 3 made no mention of cell sources.

Marketing strategies to become a stem cell doctor

We analyzed marketing language among training organizations. Six training organizations mentioned they would assist learners in marketing and growing their practice with stem cell therapies and/or increasing revenue (Table 1). Eight organizations included learner testimonials as text and/or video, most of which were created by physicians, and all expressed a positive tone.

Instructor credentials and analysis of affiliated clinics

Eight courses (57%) among 7 organizations listed 172 instructors ranging from 4 to 71 instructors per course. Of the 172 instructors, 115 were physicians (MD and DO) with other instructors including doctor of acupuncture and oriental medicine, nurse practitioners, dentists, naturopathic doctors, dieticians and nutritionists, pharmacists, industry representatives,

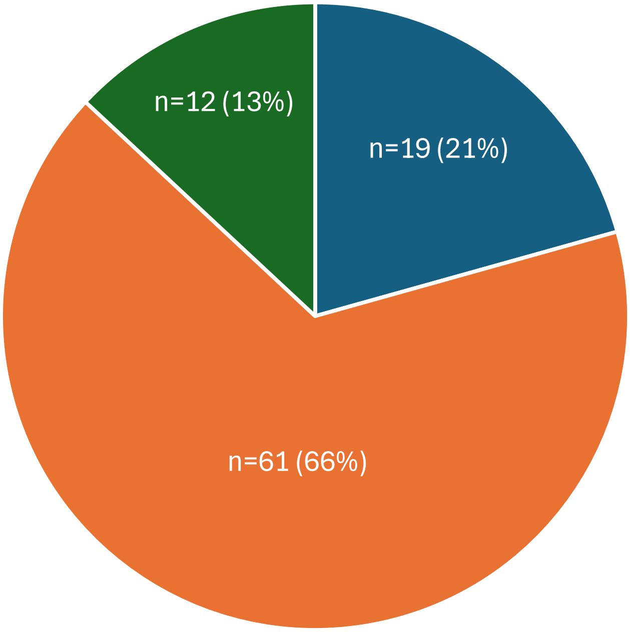

Figure 1. Instructor-affiliated clinics providing unproven, questionable, and proven treatments

A total of 92 instructor-affiliated clinics were identified. Unproven therapies, orange; questionable therapies, green; proven therapies, dark blue.

Drug Enforcement Administration inspectors, and medical coders. Specialties of physicians are reported in Table S2 (supplemental information).

Of the 115 physician instructors, we were able to identify 92 affiliated clinics. Using Google searches, we were able to determine whether the clinic affiliated with the instructor offered unproven, questionable, or proven treatments that were verified by a second coder. Unproven therapies were defined as lacking tier 1 clinical data to demonstrate clinical efficacy of the product, had expert opinion(s) suggesting the therapy should not be offered, and/or a Food and Drug Administration (FDA) warning. Questionable therapies had conflicting clinical data on treatment safety and/or efficacy and lacked consensus among scientific experts as to whether the therapy should be offered. Clinics offering both questionable and unproven interventions were coded as providing unproven therapies. Proven therapies were current standard of care or had FDA approval (see supplemental information).

Among the 92 instructor-affiliated clinics, most clinics (61, 66%) offered unproven therapies (e.g., intravenous vitamins, ozone therapy, platelet-rich plasma for autism) and 12 clinics (13%) offered questionable therapies (e.g., bioidentical hormone replacement therapy). Only 19 clinics (24%) offered proven therapies (Figure 1). Among clinics offering 1 or more unproven therapies, almost half (29, 48%) provided 3–5 unproven therapies, 21 clinics (35%) provided 1–2 unproven therapies, and 10 clinics (17%) provided 6 or more unproven therapies.

Red flags for physicians considering stem cell training

At present, there is limited training available for physicians interested in stem cell and regenerative therapy. Our analysis suggests that the training being offered might present a false impression to learners that stem cells "can" be incorporated into clinical practice. This could have significant downstream effects in perpetuating misinformation about unproven SCIs and potentially harming patients, thus contributing to the already decreasing trust of physicians and the medical establishment. Our data suggest that illegitimate stem cell training programs use similar strategies to clinics engaged in the direct-to-consumer marketing of unproven SCIs in an attempt to showcase legitimacy of their training program. Practitioners may use such courses as tokens of scientific legitimacy to provide unproven SCIs to patients (Sipp et al., 2017). Physicians interested in learning more about stem cells and their potential incorporation into the clinic should aim to identify reputable training programs and look out for red flags. We highlight five red flags based on our data that physicians should reflect on when considering a stem cell training program.

The first red flag is to avoid training programs that use hyped language that promote the idea that SCIs can currently be incorporated into medical practice and that the learner would increase their revenue. This language could be seen as part of online marketing or among learner testimonials. For example, some programs use language that, upon completion of the training, the learner would be able to deliver SCIs to patients and no additional training is necessary or that physicians can be permitted to offer SCIs and they just need to be trained on how to do them. This message clearly conveyed by multiple national and international scientific bodies is that stem cell science is in preclinical or clinical phases of research and should not be offered to patients outside of a clinical trial (Lovell-Badge et al., 2021; Ikonomou et al., 2023).

A second red flag is having a narrow scope of training in an education program. While it is beyond the scope of this article to outline key topics for

training physicians, it is likely that training medical professionals would cover a breadth of topics including stem cell biology, responsible clinical translation, regulations and policy, bioethics, patient communication, and clinical practice (Wyles et al., 2019). Courses focusing solely on stem cell therapy for practical application and providing practicums on how to administer stem cells or other unproven interventions should be met with suspicion.

A third red flag relates to the qualifications of the instructors. Our results showed that most instructors were physicians who may not have had the specialty training to treat specific degenerative conditions as previously shown (Fu et al., 2019) or to deliver the diversity of topics necessary for a stem cell training program for clinicians. A well-structured course for medical professionals that covers a breadth of topics discussed earlier should have instructors with diverse academic backgrounds (e.g., bioethics, regulatory, business management, and clinical trial expertise) who should be affiliated with an academic institution and have the appropriate expertise and experience to offer specific pedagogy. Potential learners should be weary of courses where the content focuses only on clinical application without rigorous evidence.

A fourth red flag for learners to consider is the reputability of the organizations providing stem cell courses. It is likely that legitimate training would be offered as part of an academic residency program, medical or graduate school, or reputable specialized professional or medical organizations (i.e., a clinical society), but this is not a straightforward marker of legitimacy. Several aforementioned businesses had affiliated themselves by displaying logos of recognized universities as a token of legitimacy that is likely to confuse potential learners about the credibility of such programs. Additionally, while some training programs claimed to be “fellowships,” only few were affiliated with an academic institution or a recognized academic society, and the extent of these bodies’ involvement in the course is unclear. Interested learners should seek out training in the handful of academic institutions that explicitly delineate proven and unproven therapies and have ongoing preclinical and clinical SCI trials (Wyles et al., 2019).

A fifth and final red flag is that just because courses offer CME credit does not mean they are legitimate as our results showed that 5 of 9 businesses provided CME courses. At least 10% of CME courses are industry sponsored and could lead to significant bias in training physicians (Ranganathan and Prasad, 2023).

CONCLUSION

Physicians interested in learning more about stem cells and eventually incorporating them into practice need to be trained appropriately. Stem cell education courses for physicians in the US had marketing and revenue-generating language, and most instructors were linked to clinics administering unproven therapies.

It is unclear whether physicians interested in learning more about SCIs would be able to recognize legitimate from less legitimate educational courses and vendors easily. Our results suggest that physicians interested in training on SCIs should remain skeptical of courses using hyped language such as advertising monetary gains and the immediate incorporation of SCIs into practice. Learners should also remain skeptical of programs that do not train physicians broadly about stem cell topics, where the instructor pool is mostly of a single background, e.g., only clinicians, and where no research is conducted by instructors or participants. Finally, physicians should consider training offered by reputable medical and academic organizations.

AUTHOR CONTRIBUTIONS

L.E. made substantial contributions to the collection and analysis of data, including identifying educational programs and searching the backgrounds of instructors, codebook development and modification, and served as primary qualitative coder. L. E. helped with the inital drafting of the paper, and revised the manuscript for important intellectual content. M.A. made substantial contributions to the analysis of data serving as secondary coder and revised the manuscript for important intellectual content. Z.M. made substantial contributions to the conception and design of the research project, helped revise the codebook, wrote the initial draft of the paper, and subsequently revised it for important intellectual content based on reviewer feedback. All authors approved the final version to be published and agreed to be accountable for all aspects of the work.

DECLARATION OF INTERESTS

Z.M. is a member of the Education Committee and the Public Policy Committee of the International Society for Stem Cell Research (ISSCR). ISSCR did not play a role in the research, and the views expressed in this paper do not reflect those of ISSCR, Wake Forest University School of Medicine, Wake Forest University, and Mayo Clinic.

SUPPLEMENTAL INFORMATION

Supplemental information can be found online at https://doi.org/10.1016/j.stemcr.2025.102510.

REFERENCES

American Board of Regenerative Medicine. ABRM Board Certification Course. An in-depth module based review of regenerative medicine from the American Academy of Regenerative Medicine. https://www.ambrm.org/board-certification-course.asp.

Fu, W., Smith, C., Turner, L., Fojtik, J., Pacyna, J.E., and Master, Z. (2019). Characteristics and Scope of Training of Clinicians

Participating in the US Direct-to-Consumer Marketplace for Unproven Stem Cell Interventions. JAMA *321*, 2463–2464. https://doi.org/10.1001/jama.2019.5837.

Ikonomou, L., Cuende, N., Forte, M., Grilley, B.J., Levine, A.D., Munsie, M., Rasko, J.E.J., Turner, L., Bidkhori, H.R., Ciccocioppo, R., et al. (2023). International Society for Cell & Gene Therapy Position Paper: Key considerations to support evidence-based cell and gene therapies and oppose marketing of unproven products. Cytotherapy *25*, 920–929. https://doi.org/10.1016/j.jcyt.2023.03.002.

International Society for Stem Cell Research (2024). ISSCR Creates First-of-its Kind Continuing Education Course on Stem Cell-Based Medicine. https://www.isscr.org/isscr-news/continuing-education.

Knoepfler, P.S. (2013). Call for fellowship programs in stem cell-based regenerative and cellular medicine: a new stem cell training is essential for physicians. Regen. Med. *8*, 223–225. https://doi.org/10.2217/rme.13.1.

Lovell-Badge, R., Anthony, E., Barker, R.A., Bubela, T., Brivanlou, A.H., Carpenter, M., Charo, R.A., Clark, A., Clayton, E., Cong, Y., et al. (2021). ISSCR Guidelines for Stem Cell Research and Clinical Translation: The 2021 update. Stem Cell Rep. *16*, 1398–1408. https://doi.org/10.1016/j.stemcr.2021.05.012.

Ranganathan, S., and Prasad, V. (2023). CME: Is it Meeting the Mark? Am. J. Med. *136*, 618–619. https://doi.org/10.1016/j.amjmed.2023.02.010.

Sipp, D., Caulfield, T., Kaye, J., Barfoot, J., Blackburn, C., Chan, S., De Luca, M., Kent, A., McCabe, C., Munsie, M., et al. (2017). Marketing of unproven stem cell-based interventions: A call to action. Sci. Transl. Med. *9*, eaag0426. https://doi.org/10.1126/scitranslmed.aag0426.

Smith, C., Crowley, A., Munsie, M., DeMartino, E.S., Staff, N.P., Shapiro, S., and Master, Z. (2021). Academic physician specialists' views toward the unproven stem cell intervention industry: areas of common ground and divergence. Cytotherapy *23*, 348–356. https://doi.org/10.1016/j.jcyt.2020.12.011.

Turner, L. (2021). The American stem cell sell in 2021: U.S. businesses selling unlicensed and unproven stem cell interventions. Cell Stem Cell *28*, 1891–1895. https://doi.org/10.1016/j.stem.2021.10.008.

UCSF. Herbert Perkins Cellular Therapy & Transfusion Medicine Fellowship. https://cellulartherapyfellowship.ucsf.edu/.

University of Illinois College of Medicine Department of Ophthalmology and Visual Sciences. Ocular Regenerative Medicine Fellowship. https://chicago.medicine.uic.edu/ophthalmology-visual-sciences/education/fellowship-programs/orm-fellowship/.

University of Pennsylvania Perelman School of Medicine Pathology and Laboratory Medicine. Cellular Therapy Fellowship. https://pathology.med.upenn.edu/education/fellowships/programs/cellular-therapy-fellowship.

USC Kerk School of Medicine (2024). Call for Applications: CIRM Clinical Research Fellowship in Stem Cell Biology and Regenerative Medicine. https://stemcell.keck.usc.edu/2024-call-for-applications-cirm-clinical-research-fellowship/.

Wyles, S.P., Hayden, R.E., Meyer, F.B., and Terzic, A. (2019). Regenerative medicine curriculum for next-generation physicians. NPJ Regen. Med. *4*, 3. https://doi.org/10.1038/s41536-019-0065-8.

Stem Cell Reports, Volume *20*

Supplemental Information

An analysis of stem cell training programs for physicians in the US—A mirage of credibility

Luqman Ellythy, Mohamed Addani, and Zubin Master

SUPPLEMENTAL INFORMATION

Stem Cell Reports 2025

An Analysis of Stem Cell Training Programs for Physicians in the U.S. – A Mirage of Credibility

Luqman Ellythy, Mohamed Addani, Zubin Master

Supplemental Methods

A google search of stem cell therapy educational courses for physicians was conducted in August 2022 using the terms "physician" or "doctor," and "course" or "class" or "training," and "stem cell therapy" or "regenerative medicine." We included training websites offering onsite, online, or hybrid training targeted to physicians or other clinicians. Our selection of training programs targeted practicing physicians or other clinicians where the course aimed to promote the practice of unproven stem cell intervention (SCIs). We excluded training websites that were not written in English, where the primary target was not practicing physicians or other clinicians, and programs with only onsite training that was not offered in the U.S. These criteria excluded MS and PhD programs surrounding stem cell laboratory or clinical research. We excluded residency and fellowship programs offered by university-affiliated hospitals and academic medical centers, and training offered by reputable clinical societies and other professional bodies. All course websites meeting the inclusion criteria were systematically analyzed by a single coder using the codebook below. A second coder validated the primary coder's data. Any discrepancies among coders were discussed and a mutual decision was reached.

Codebook Development:

The codebook was developed to analyze the nature of regenerative medicine training for physicians and other clinicians. The codebook was developed based on previously conducted research and inductively, based on an analysis of 3 training programs. The codebook was modified after inductive assessment of the 3 training programs and then systematically applied to all training websites meeting the inclusion criteria. The codebook contained 4 parts: 1) course features; 2) marketing language; and 3) instructors and their affiliations.

Final Codebook

1) Marketing Language:

We analyzed all websites within the domain of the training program for marketing language excluding learner testimonials.

- Included major themes related to the mission statement as a qualitative description and record exact quotes as examples
- Examples of hyped marketing language include: helping incorporate stem cells in practice, increase clinic profitability

2) Course Content

We collected course type, course name, number of hours, location, cost, type of training offered (e.g., regenerative medicine, aesthetic, marketing), mode of training (online, hybrid, or onsite), practicum description (if applicable), affiliation with external organizations (e.g., clinics, academic institutions), subject topics covered, mission statement, and instructor and learner testimonials.

Hours: Is the number of hours needed to complete the course indicated? (Y/N)

- If yes, include number of hours or describe the length of the course generally i.e., days, weeks, year.
- If yes, note the general timeframe given. If no, note if it is an online course to be taken at your own pace.

Mode of Training: Indicate the mode of training for this course: Online, Hybrid, or In-Person

Practicum: Does the course have a practicum (Y/N)?

- If yes, include a qualitative description of practicum

Onsite Location: Does the course have an onsite training component? (Y/N)

- If yes, include a qualitative description of the location of the onsite component

Cost: Is the cost of the course indicated without needing to sign up or email for more information? (Y/N)

- If yes, indicate the total cost of course

Continuing Medical Education (CME) Accreditation: Did the business mention having CME accreditation? Y/N)

Use of Autologous/Allogenic Cell Sources: Did the business mention anywhere that learners will receive training with autologous cell sources, allogenic cell sources, both, or did not mention?

Organization/Business Who Offers the Course(s): Include a qualitative description of the organization or business offering the course

- This may be the name of course itself
- Examine “About Us” statement, or objective(s) of the training program

Affiliations: Did the course list affiliated external partner(s)? (Y/N)

- If yes, provide a qualitative description of all listed affiliations
- Affiliated partners may include another business, organization, biotech company, universities, or academic institutions

Topics: Are the topics/subjects of the course covered?

- If yes, include a qualitative description of all the major topics/subjects covered

Outside References: Was there any statement/inference to an outside institution that provides stem cell treatment? (Y/N)

- Exclude instructor biographies
- If yes, include the name of the institution e.g., Mayo Clinic, name of unproven stem cell clinic

Learner Testimonials: Are there testimonials present on the website page? (Y/N)

- If yes, what format are they in? (Written, Videos or both)

3) Nature of Therapies Provided by Instructors at Their Affiliated Clinics:
For courses listing the names of instructors, we cross referenced the names with outside clinics via Google searches to evaluate the types of therapies instructors offered. We used the term “stem cell” with names to help identify instructors. In cases where additional clinic affiliation of the instructor was provided on the course website, we also searched the name of the instructor with the name of the clinic. We also cross-referenced the instructor’s name with the geographical location of their clinic if mentioned in their biography. We evaluated each instructor in terms of whether they provided treatments categorized as *unproven*, *questionable*, or *proven*.

Therapies classified as *unproven* were those that did not have any randomized controlled trials demonstrating treatment efficacy and lacked Tier 1 clinical data. If clinical studies were performed, we checked UpToDate, specialty guidelines from relevant clinical societies, and for the presence of an FDA warning to indicate whether the therapy should or should not be offered. Therapies classified as *questionable* included those with conflicting clinical data of treatment safety and/or efficacy and expert opinions in the academic literature were inconclusive as to whether the therapy should be offered. No clinic websites explicitly stated that the *unproven* or *questionable* therapies were given as part of a clinical trial, and thus *unproven* and *questionable* therapies were coded as “unproven” or questionable” respectively based on the above criteria. While websites did not explicitly mention whether *unproven* or *questionable* therapies were part of clinical research, this assumption constitutes a limitation of website content analysis. Therapies classified as *proven* only offered standard-of-care treatments or FDA approved treatments. Clinics classified as offering only *proven* therapies cannot advertise offering *unproven* or *questionable* therapies. A clinic would be counted as offering only *questionable* therapies if they offered no *unproven* therapies. Clinics offering both *questionable* and *unproven* therapies would be coded as providing *unproven*. The *unproven*, *questionable*, and *proven* categories were classified to clinics because more than 1 instructor (or other practitioner) was associated with a single clinic and we cannot be certain who among them was offering the *unproven*, *questionable*, or *proven* therapies (Fu et al., 2019 JAMA). A physician trainee (LE) determined whether therapies were classified as unproven, questionable, or proven and sought advice from senior Mayo Clinic physician experts as needed.

Are the instructors listed on the website and clearly indicated as instructor or team? (Y/N)

- If yes list the number of instructors
- Identify the backgrounds of all instructors
- For physician instructors with MD or DO, identify areas of residency or fellowship specialty training.

Unproven & Questionable Therapies:

- For each instructor-affiliated clinic, write down the number of unproven and questionable therapies provided for specific indications. Application of an unproven/questionable therapy for each indication listed is counted except IV vitamins (see Table S1)

Proven Therapies:
There is no evidence of the instructor providing unproven or questionable therapies in affiliated clinic.

- Counted as 0 for the number of unproven therapies provided.

Table S1: Level of evidence for unproven and questionable therapies offered at instructor-affiliated clinics

Treatment	Number of Clinics	Indication	Classification	References
Hyperbaric Oxygen	5	Anti-aging	Unproven	1
Ozone	7	Anti-aging	Unproven	2
Bioidentical Hormone Replacement Therapy	18	Anti-aging, increased energy	Questionable	3,4
NAD+ IV therapy	2	Anti-aging	Unproven	5,6
Vampire Needling + platelet-rich plasma (PRP) for face	20	Skin rejuvenation	Unproven	7
IV vitamins: Myer's cocktail, Curcumin, Vitamin C.	25	Antiaging, cancer therapy	Unproven	8, 9, 43
Infrared and red-light sauna	2	Energy and antiaging	Unproven	10, 39
Activated Air Therapy/singlet oxygen therapy	1	Biohacking (antiaging)	Unproven	11
Vitamin B12 injections	1	Weight loss	Unproven	12
VSELs (very small embryonic-like stem cells)	1	Decreased joint pain, increased energy, improved metabolic function, and improved cognitive function	Unproven	13
pH manipulation therapy	2	Cancer	Unproven	14
Prolotherapy	1	Chronic pain	Questionable	15, 16
Prolozone	6	Chronic neck and back pain + joint arthritis	Unproven	17
Therapeutic ultrasound	3	Healing Injuries. Improve pain, function and range of motion.	Unproven	18
Photoactivated PRP	1	"Healing"	Unproven	19
P shots (priapism shots)	11	Erectile Dysfunction	Unproven	20
O shots (orgasm shots)	10	Vaginal rejuvenation and sexual dysfunction	Unproven	21

HCG (human chorionic gonadotropin) diet	10	Weight loss	Unproven	22
NO therapy	1	Sexual dysfunction	Unproven	23
Total nonspecific PRP	10	No Indication	Unproven	
PRP	5	Autism	Unproven	24
PRP	8	MS	Unproven	25 26
PRP	9	COPD	Unproven	27 28 no RCTs
PRP	1	Heart Failure	Unproven	29
PRP	10	joints/arthritis	Questionable	31
TMS (transcranial magnetic stimulation)	1	Autism	Unproven	30
Stem cells	10	orthobiologics	Unproven	32, 33
Extracorporeal shock therapy	2	Tendinopathy	Questionable	33
Amniotic stem cells	3		Unproven	34
PRP	5	Hair restoration	Questionable	52
Thread embedding Therapy	5	Hair restoration	Unproven	7
Therapeutic plasma exchange	1	Anti-aging	Unproven	35
Homeopathy	1	Any specific indication	Unproven	47
Laser therapy	2	Tendinopathy	Unproven	36
Alpha 2 Macroglobulin (A2M) Injections	5	Ortho	Unproven	37
Peptide therapy	5	Cancer	Unproven	46
Tenex	10	Tendinopathy	Unproven	45
Reflexotherapy	1	cancer	Unproven	38
Whole body cryotherapy	1	Weight loss and fibromyalgia	Unproven	40, 41
Vaginal rejuvenation radiofrequency	1	Sexual Function	Unproven	42
Lonator body cleanse	1	Toxin removal	Unproven	44
IV ketamine	1	Wellness and fatigue	Unproven	49
Hydropathy	1	Pain	Unproven	50
Quantum Therapy	1	Pain	Unproven	51

Among the 115 physician instructors, we documented their specialty training (Table S2).

Table S2. Residency and Fellowship Specialties Among Physician Instructors

Specialty	Number of Physician Instructors
Aesthetics	1
Allergy and Immunology	1
Anesthesiology	5
Cardiology	4
Dermatology	6
Emergency Medicine	6
Endocrinology	1
Ear, Nose and Throat	2
Facial Plastics	2
Family Medicine	15
General Surgery	5
Geriatrics	1
Gynecology-Oncology	1
Internal Medicine	9
Neurology	2
Neurosurgery	2
Obstetrics and Gynecology	6
Occupational and Environmental Medicine	1
Oncology	2
Ophthalmology	1
Oral Maxillofacial Surgery	1
Orthopedics	4
Pain	3
Pathology	1
Pediatrics	2
Physical Medicine and Rehabilitation	2
Plastic Surgery	5
Preventative Medicine	1
Psychiatry	3
Radiology	1
Spine (Orthopedics)	1
Sports Medicine	14
Unknown	2
Urology	2

References:

1. Jones MW, Cooper JS. Hyperbaric Therapy for Wound Healing. [Updated 2023 Jun 12]. In: StatPearls [Internet]. Treasure Island (FL): StatPearls Publishing; 2024 Jan-. Available from: https://www.ncbi.nlm.nih.gov/books/NBK459172/
2. U.S. Food and Drug Administration. (n.d.). 21 CFR § 801.415 - Labeling for in vitro diagnostic devices. Retrieved August 13, 2024, from https://www.accessdata.fda.gov/scripts/cdrh/cfdocs/cfcfr/cfrsearch.cfm?fr=801.415
3. U.S. Food and Drug Administration. (n.d.). National Academies of Sciences, Engineering, and Medicine (NASEM) study on the clinical utility of treating patients. U.S. Food and Drug Administration. Retrieved August 13, 2024, from https://www.fda.gov/drugs/human-drug-compounding/national-academies-science-engineering-and-medicine-nasem-study-clinical-utility-treating-patients
4. National Academies of Sciences, Engineering, and Medicine. (2019). The clinical utility of treating patients with antimicrobial agents. In The clinical utility of treating patients with antimicrobial agents (Chapter 9). National Academies Press. https://nap.nationalacademies.org/read/25791/chapter/9#166
5. Braidy N, Liu Y. NAD+ therapy in age-related degenerative disorders: A benefit/risk analysis. Exp Gerontol. 2020 Apr;132:110831. doi: 10.1016/j.exger.2020.110831. Epub 2020 Jan 7. PMID: 31917996.
6. Radenkovic D, Reason, Verdin E. Clinical Evidence for Targeting NAD Therapeutically. Pharmaceuticals (Basel). 2020 Sep 15;13(9):247. doi: 10.3390/ph13090247. PMID: 32942582; PMCID: PMC7558103.
7. Gupta AK, Versteeg SG, Rapaport J, Hausauer AK, Shear NH, Piguet V. The Efficacy of Platelet-Rich Plasma in the Field of Hair Restoration and Facial Aesthetics-A Systematic Review and Meta-analysis. J Cutan Med Surg. 2019 Mar/Apr;23(2):185-203. doi: 10.1177/1203475418818073. Epub 2019 Jan 4. PMID: 30606055.
8. Moertel CG, Fleming TR, Creagan ET, Rubin J, O'Connell MJ, Ames MM. High-dose vitamin C versus placebo in the treatment of patients with advanced cancer who have had no prior chemotherapy. A randomized double-blind comparison. N Engl J Med. 1985 Jan 17;312(3):137-41. doi: 10.1056/NEJM198501173120301. PMID: 3880867.
9. Nelson, K. M., Dahlin, J. L., Bisson, J., Graham, J., Pauli, G. F., & Walters, M. A. (2017). The Essential Medicinal Chemistry of Curcumin. Journal of medicinal chemistry, 60(5), 1620–1637. https://doi.org/10.1021/acs.jmedchem.6b00975
10. Beever R. Far-infrared saunas for treatment of cardiovascular risk factors: summary of published evidence. Can Fam Physician. 2009 Jul;55(7):691-6. PMID: 19602651; PMCID: PMC2718593.
11. Li, X., Lovell, J. F., Yoon, J., & Chen, X. (2020). Clinical development and potential of photothermal and photodynamic therapies for cancer. Nature reviews. Clinical oncology, 17(11), 657–674. https://doi.org/10.1038/s41571-020-0410-2
12. Boachie, J., Adaikalakoteswari, A., Samavat, J., & Saravanan, P. (2020). Low Vitamin B12 and Lipid Metabolism: Evidence from Pre-Clinical and Clinical Studies. Nutrients, 12(7), 1925. https://doi.org/10.3390/nu12071925
13. Ratajczak, M. Z., Ratajczak, J., & Kucia, M. (2019). Very Small Embryonic-Like Stem Cells (VSELs). Circulation research, 124(2), 208–210. https://doi.org/10.1161/CIRCRESAHA.118.314287

14. 1Yang M, Zhong X, Yuan Y. Does Baking Soda Function as a Magic Bullet for Patients With Cancer? A Mini Review. Integrative Cancer Therapies. 2020;19. doi:10.1177/1534735420922579
15. Morath, O., Kubosch, E. J., Taeymans, J., Zwingmann, J., Konstantinidis, L., Südkamp, N. P., & Hirschmüller, A. (2018). The effect of sclerotherapy and prolotherapy on chronic painful Achilles tendinopathy-a systematic review including meta-analysis. Scandinavian journal of medicine & science in sports, 28(1), 4–15. https://doi.org/10.1111/sms.12898
16. Taheem, Y. and Suvar, T. (2021) Prolotherapy: Review with background history, mechanism of action, and current evidence, ASRA Pain Medicine. Available at: https://www.asra.com/news-publications/asra-updates/blog-landing/legacy-b-blog-posts/2021/10/15/prolotherapy-review-with-background-history-mechanism-of-action-and-current-evidence (Accessed: 13 August 2024).
17. CFR - Code of Federal Regulations Title 21 (no date) accessdata.fda.gov. Available at: https://www.accessdata.fda.gov/scripts/cdrh/cfdocs/cfcfr/cfrsearch.cfm?fr=801.415#:~:text=801.415%20Maximum%20acceptable%20level%20of,tolerated%20by%20man%20and%20animals. (Accessed: 13 August 2024).
18. Miller DL, Smith NB, Bailey MR, Czarnota GJ, Hynynen K, Makin IR; Bioeffects Committee of the American Institute of Ultrasound in Medicine. Overview of therapeutic ultrasound applications and safety considerations. J Ultrasound Med. 2012 Apr;31(4):623-34. doi: 10.7863/jum.2012.31.4.623. PMID: 22441920; PMCID: PMC3810427.
19. Paterson, K. L., Nicholls, M., Bennell, K. L., & Bates, D. (2016). Intra-articular injection of photo-activated platelet-rich plasma in patients with knee osteoarthritis: a double-blind, randomized controlled pilot study. BMC musculoskeletal disorders, 17, 67. https://doi.org/10.1186/s12891-016-0920-3
20. Burnett, A. L., Nehra, A., Breau, R. H., Culkin, D. J., Faraday, M. M., Hakim, L. S., Heidelbaugh, J., Khera, M., McVary, K. T., Miner, M. M., Nelson, C. J., Sadeghi-Nejad, H., Seftel, A. D., & Shindel, A. W. (2018). Erectile Dysfunction: AUA Guideline. The Journal of urology, 200(3), 633–641. https://doi.org/10.1016/j.juro.2018.05.004
21. Stanley, E. E., & Pope, R. J. (2022). Characteristics of Female Sexual Health Programs and Providers in the United States. Sexual medicine, 10(4), 100524. https://doi.org/10.1016/j.esxm.2022.100524
22. Commissioner, O. of the (2020) Avoid dangerous HCG diet products, U.S. Food and Drug Administration. Available at: https://www.fda.gov/consumers/consumer-updates/avoid-dangerous-hcg-diet-products (Accessed: 13 August 2024).
23. Burnett AL. The role of nitric oxide in erectile dysfunction: implications for medical therapy. J Clin Hypertens (Greenwich). 2006 Dec;8(12 Suppl 4):53-62. doi: 10.1111/j.1524-6175.2006.06026.x. PMID: 17170606; PMCID: PMC8109295.
24. Ardhanareeswaran K, Coppola G, Vaccarino F. The use of stem cells to study autism spectrum disorder. Yale J Biol Med. 2015 Mar 4;88(1):5-16. PMID: 25745370; PMCID: PMC4345539.
25. Farid MF, Abouelela YS, Yasin NAE, Mousa MR, Ibrahim MA, Prince A, Rizk H. A novel cell-free intrathecal approach with PRP for the treatment of spinal cord multiple sclerosis in cats. Inflamm Regen. 2022 Oct 14;42(1):45. doi: 10.1186/s41232-022-00230-w. PMID: 36229845; PMCID: PMC9563497.

26. Borhani-Haghighi, M., & Mohamadi, Y. (2019). The therapeutic effect of platelet-rich plasma on the experimental autoimmune encephalomyelitis mice. Journal of neuroimmunology, 333, 476958. https://doi.org/10.1016/j.jneuroim.2019.04.018
27. Mammoto T, Chen Z, Jiang A, Jiang E, Ingber DE, Mammoto A. Acceleration of Lung Regeneration by Platelet-Rich Plasma Extract through the Low-Density Lipoprotein Receptor-Related Protein 5-Tie2 Pathway. Am J Respir Cell Mol Biol. 2016 Jan;54(1):103-13. doi: 10.1165/rcmb.2015-0045OC. PMID: 26091161; PMCID: PMC5455682.
28. Knight AD, Kacker S. Platelet-Rich Plasma Treatment for Chronic Respiratory Disease. Cureus. 2023 Jan 2;15(1):e33265. doi: 10.7759/cureus.33265. PMID: 36741673; PMCID: PMC9891651.
29. Spartalis E, Tomos P, Moris D, Athanasiou A, Markakis C, Spartalis MD, Troupis T, Dimitroulis D, Perrea D. Role of platelet-rich plasma in ischemic heart disease: An update on the latest evidence. World J Cardiol. 2015 Oct 26;7(10):665-70. doi: 10.4330/wjc.v7.i10.665. PMID: 26516421; PMCID: PMC4620078.
30. Shen, Y. X., Fan, Z. H., Zhao, J. G., & Zhang, P. (2009). The application of platelet-rich plasma may be a novel treatment for central nervous system diseases. Medical hypotheses, 73(6), 1038–1040. https://doi.org/10.1016/j.mehy.2009.05.021
31. Weissman, L. and Harris, H.K. (2024) Autism spectrum disorder in children and adolescents: Complementary and alternative therapies, UpToDate. Available at: https://www.uptodate.com/contents/autism-spectrum-disorder-in-children-and-adolescents-complementary-and-alternative-therapies?search=MeRT+TMS&source=search_result&selectedTitle=10~150&usage_type=default&display_rank=10#H2917575028 (Accessed: 13 August 2024).
32. O'Dowd A. Update on the Use of Platelet-Rich Plasma Injections in the Management of Musculoskeletal Injuries: A Systematic Review of Studies From 2014 to 2021. Orthopaedic Journal of Sports Medicine. 2022;10(12). doi:10.1177/23259671221140888
33. Young, M, Dijkstra, P, Biologic therapies for tendon and muscle injury UpToDate. (2024). Retrieved August 13, 2024, from Uptodate.com website: https://www.uptodate.com/contents/biologic-therapies-for-tendon-and-muscle-injury?search=prgf&source=search_result&selectedTitle=1~65&usage_type=default&display_rank=1#H2827233597
34. Purdam, CR, UpToDate. (2024). Retrieved August 13, 2024, from Uptodate.com website: https://www.uptodate.com/contents/overuse-persistent-tendinopathy-overview-of-management#H3483872523
35. Suh, S., Yale, K. L., & Mesinkovska, N. A. (2021). 28383 The effectiveness of thread-embedding therapy for treating scarring and nonscarring alopecia. Journal of the American Academy of Dermatology, 85(3), AB186–AB186. https://doi.org/10.1016/j.jaad.2021.06.757
36. Hunter M. Use of homeopathy in NHS not justified. BMJ. 2002 Mar 9;324(7337):565. PMCID: PMC1172082.
37. Tumilty, S., Munn, J., McDonough, S., Hurley, D. A., Basford, J. R., & Baxter, G. D. (2010). Low level laser treatment of tendinopathy: a systematic review with meta-analysis. Photomedicine and laser surgery, 28(1), 3–16. https://doi.org/10.1089/pho.2008.2470
38. Vajapey, S., Ghenbot, S., Baria, M. R., Magnussen, R. A., & Vasileff, W. K. (2021). Utility of Percutaneous Ultrasonic Tenotomy for Tendinopathies: A Systematic Review. Sports health, 13(3), 258–264. https://doi.org/10.1177/1941738120951764

39. Ernst, E., P Posadzki, & Lee, M. S. (2011). Reflexology: An update of a systematic review of randomised clinical trials. Maturitas, 68(2), 116–120. https://doi.org/10.1016/j.maturitas.2010.10.011
40. Glass G. E. (2021). Photobiomodulation: The Clinical Applications of Low-Level Light Therapy. Aesthetic surgery journal, 41(6), 723–738. https://doi.org/10.1093/asj/sjab025
41. Hernández-Bule ML, Naharro-Rodríguez J, Bacci S, Fernández-Guarino M. Unlocking the Power of Light on the Skin: A Comprehensive Review on Photobiomodulation. Int J Mol Sci. 2024 Apr 19;25(8):4483. doi: 10.3390/ijms25084483. PMID: 38674067; PMCID: PMC11049838.
42. Whole body cryotherapy can be hazardous to your skin (no date) American Academy of Dermatology. Available at: https://www.aad.org/public/cosmetic/safety/cryotherapy (Accessed: 13 August 2024).
43. Commissioner, O. of the (no date) Statement from FDA commissioner Scott Gottlieb, M.D., on efforts to safeguard women's health from deceptive health claims and significant risks related to devices marketed for use in medical procedures for 'vaginal rejuvenation', U.S. Food and Drug Administration. Available at: https://www.fda.gov/news-events/press-announcements/statement-fda-commissioner-scott-gottlieb-md-efforts-safeguard-womens-health-deceptive-health-claims (Accessed: 13 August 2024).
44. Ali, A., Njike, V. Y., Northrup, V., Sabina, A. B., Williams, A. L., Liberti, L. S., Perlman, A. I., Adelson, H., & Katz, D. L. (2009). Intravenous micronutrient therapy (Myers' Cocktail) for fibromyalgia: a placebo-controlled pilot study. Journal of alternative and complementary medicine (New York, N.Y.), 15(3), 247–257. https://doi.org/10.1089/acm.2008.0410
45. Barman P, Joshi S, Sharma S, Preet S, Sharma S, Saini A. Strategic Approaches to Improvise Peptide Drugs as Next Generation Therapeutics. Int J Pept Res Ther. 2023;29(4):61. doi: 10.1007/s10989-023-10524-3. Epub 2023 May 24. PMID: 37251528; PMCID: PMC10206374.
46. Castro, J. C., Wang, D., & Chien, G. C. C. (2022). Regenerative medicine for neuropathic pain: physiology, ultrasound and therapies with a focus on alpha-2-macroglobulin. Pain management, 12(6), 779–793. https://doi.org/10.2217/pmt-2022-0006
47. Fernández-Zarzoso, M., Gómez-Seguí, I., & de la Rubia, J. (2019). Therapeutic plasma exchange: Review of current indications. Transfusion and apheresis science : official journal of the World Apheresis Association : official journal of the European Society for Haemapheresis, 58(3), 247–253. https://doi.org/10.1016/j.transci.2019.04.007
48. Center. (2024). Important Patient and Consumer Information About Regenerative Medicine. Retrieved August 14, 2024, from U.S. Food and Drug Administration website: https://www.fda.gov/vaccines-blood-biologics/consumers-biologics/important-patient-and-consumer-information-about-regenerative-medicine-therapies
49. Thase, M. and Connolly, R. (2024) Ketamine and esketamine for treating unipolar depression in adults: Administration, efficacy, and adverse effects, UpToDate. Available at: https://www.uptodate.com/contents/ketamine-and-esketamine-for-treating-unipolar-depression-in-adults-administration-efficacy-and-adverse-effects?search=ketamine&source=search_result&selectedTitle=2~150&usage_type=default&display_rank=1 (Accessed: 27 August 2024).
50. Ortiz M, Koch AK, Cramer H, Linde K, Rotter G, Teut M, Brinkhaus B, Haller H. Clinical effects of Kneipp hydrotherapy: a systematic review of randomised controlled trials. BMJ Open. 2023 Jul 9;13(7):e070951. doi: 10.1136/bmjopen-2022-070951. PMID: 37423627; PMCID: PMC10335435.

51. Barassi G, Pokorski M, Pellegrino R, Supplizi M, Prosperi L, Marinucci C, Di Simone E, Mariani C, Younes A, Di Iorio A. Quantum Medicine: A Role of Extremely Low-Frequency Magnetic Fields in the Management of Chronic Pain. Adv Exp Med Biol. 2022;1375:23-28. doi: 10.1007/5584_2021_697. PMID: 35038149.
52. U.S. Food & Drug Administration. 2021. Important patient and consumer information about regenerative medicine therapies. https://www.fda.gov/vaccines-blood-biologics/consumers-biologics/important-patient-and-consumer-information-about-regenerative-medicine-therapies.
